# Whole-body vs. selective head cooling and target temperature strategies for neonatal HIE: a meta-analysis of long-term outcomes

**DOI:** 10.3389/fped.2026.1758783

**Published:** 2026-03-23

**Authors:** Yuping Lin, Lifeng Hu, Libin Xie, Zihui Wei

**Affiliations:** 1 Neonatology Department, Fuzhou Second General Hospital, Fuzhou, Fujian, China; 2 Neonatology Department, Fuzhou Second General Maternity and Child Health Care Hospital, Fuzhou, Fujian, China

**Keywords:** long-term outcomes, moderate, neonatal hypoxic-ischemic encephalopathy, severe, therapeutic hypothermia

## Abstract

**Objective:**

To systematically evaluate the differential long-term outcomes—including mortality, severe disability, and specific neurodevelopmental domains (language, learning, memory)—associated with various therapeutic hypothermia strategies for moderate-to-severe neonatal hypoxic-ischemic encephalopathy (HIE) through a comprehensive review and meta-analysis.

**Methods:**

A systematic search was conducted across PubMed, Embase, and the Cochrane Library up to December 2023, supplemented by gray literature and reference tracking. Randomized controlled trials (RCTs) and prospective cohort studies involving neonates ≥36 weeks' gestation with moderate-to-severe HIE treated with therapeutic hypothermia were included. Data extraction covered study characteristics, outcome data, and risk of bias indicators. The subgroup analysis, sensitivity analysis, and assessment of publication bias were carried out.

**Results:**

A total of 11 studies involving 1,466 neonates were included. The pooled composite endpoint rate (mortality + severe disability) after therapeutic hypothermia was 39.2% (95% CI: 32.7%–46.1%). Subgroup analysis revealed that whole-body cooling demonstrated superior efficacy compared to selective head cooling (RR = 0.71, *P* = 0.03), and lower target temperatures (≤34 °C) further improved outcomes (*P* = 0.04). Limited domain-specific data showed receptive language delays (18%), expressive language delays (22%), and working memory impairments (15%) in survivors without severe disability. Studies with >18-month follow-up showed a non-significant trend toward higher disability detection. The overall quality of evidence was medium, with most studies having a moderate risk of bias and low applicability concerns. No significant publication bias was detected (*P* = 0.08).

**Conclusion:**

Therapeutic hypothermia reduces the combined risk of mortality and severe disability in moderate-to-severe neonatal HIE. Whole-body cooling and target temperatures of 33–34 °C may optimize outcomes, but extreme hypothermia (≤32 °C) is associated with increased mortality, necessitating careful monitoring of adverse events. Further research is needed to optimize protocols and characterize neurodevelopmental outcomes in specific domains (e.g., language, memory).

## 1 Introduction

Neonatal hypoxic-ischemic encephalopathy (HIE) is a critical condition in neonatology, posing a substantial threat to newborns' health worldwide (1). It occurs when the neonatal brain experiences oxygen and blood flow deprivation during the perinatal period, often due to maternal complications like pre-eclampsia or intrapartum issues such as prolonged labor (2). The pathophysiology of HIE is intricate. The initial hypoxia-ischemia triggers an energy crisis in brain cells. With energy depletion, ion pumps fail, leading to calcium influx. This activates enzymes that break down cellular components, causing cell damage (3). Subsequently, in the secondary energy failure phase, excitatory neurotransmitters like glutamate are released. Excessive glutamate activates NMDA receptors, leading to more calcium influx and the production of reactive oxygen species (ROS) (4). These ROS damage cellular structures, and the activated immune system releases inflammatory cytokines, further exacerbating brain injury (5). The long-term consequences of HIE are severe. Affected neonates face a high risk of neurological disabilities, including not only cerebral palsy, intellectual impairment, and epilepsy, but also specific deficits in language (e.g., expressive/receptive language delays), learning (e.g., academic performance), and memory (e.g., working memory) that may persist into childhood, adolescence, and adulthood (6). Cerebral palsy impairs movement, while intellectual impairment affects cognitive functions. Epilepsy endangers physical safety and impacts social and emotional well-being. These disabilities burden families and healthcare systems (7).

Given the gravity of HIE, finding effective treatment strategies is crucial. Over time, multiple approaches have been explored, including pharmacological agents and stem cell therapy (8). Therapeutic hypothermia has emerged as a promising option. The principle behind it is that lowering body temperature slows the brain's metabolic rate. This reduces the demand for oxygen and glucose, inhibits the production of ROS and toxic metabolites, and suppresses cell-death pathways and the inflammatory response (9). Several clinical trials have evaluated therapeutic hypothermia for HIE. Some have reported positive outcomes, indicating a reduced risk of mortality and severe neurological disability (10). However, many uncertainties persist. There are questions about the best cooling modality (whole-body vs. selective head cooling), the optimal target temperature, the duration of cooling, and the long-term effects. Furthermore, existing studies show heterogeneity in results, which may be due to differences in patient populations, treatment protocols, and outcome assessment methods (11). A comprehensive systematic review and meta-analysis are thus necessary to synthesize the available evidence. While therapeutic hypothermia is now firmly established as the standard of care for moderate-to-severe HIE, clinical practice remains heterogeneous—with variations in cooling modality (whole-body vs. selective head cooling), target temperature (33–35.5 °C), and duration. These differences may impact long-term neurodevelopmental outcomes, yet a quantitative synthesis of their effects is lacking. This study aims to conduct a systematic review and meta-analysis to synthesize the available evidence on the differential long-term outcomes associated with different therapeutic hypothermia strategies (e.g., cooling modality, target temperature). Notably, we address critical gaps in prior meta-analyses by: (1) stratifying outcomes by HIE severity (moderate vs. severe); (2) analyzing mortality and severe disability as independent endpoints; and (3) exploring efficacy variations across economic development settings. These analyses provide novel granularity to inform personalized cooling protocols, thereby guiding the optimization of clinical practice.

## 2 Methods

### 2.1 Study design and registration

This systematic review and meta-analysis followed the Preferred Reporting Items for Systematic Reviews and Meta-Analyses (PRISMA) guidelines with registration number INPLASY2025120070.

### 2.2 Literature search strategy

A systematic search was conducted across PubMed, Embase, and the Cochrane Library up to December 2025 using Boolean operators to combine keywords: (“therapeutic hypothermia” OR “cooling”) AND (“hypoxic-ischemic encephalopathy” OR “HIE”) AND (“long-term outcomes” OR “neurodevelopment” OR “mortality” OR “disability”). The search strategy incorporated Medical Subject Headings (MeSH) and Emtree terms, supplemented by free-text variations (e.g., “whole-body cooling,” “Bayley Scales,” “cerebral palsy”), with language restrictions limited to English. Additional studies were identified through gray literature (e.g., ClinicalTrials.gov, conference abstracts) and backward reference tracking of included articles to mitigate publication bias. All retrieved records were deduplicated using EndNote and imported into Covidence for title/abstract screening and full-text eligibility assessment.

### 2.3 Eligibility criteria

**Inclusion criteria:** Population: Neonates ≥36 weeks' gestation with moderate-to-severe HIE (defined by Sarnat staging or EEG/amplitude-integrated EEG criteria). This threshold aligns with the original NICHD trials and current guidelines, which recommend TH for term neonates. Preterm infants <36 weeks were excluded due to distinct pathophysiology and limited data on TH safety/efficacy in this cohort. Studies including mild HIE were included only if data for moderate-to-severe cases were stratifiable. For studies with mixed severity, individual patient data (IPD) or subgroup data for moderate-to-severe HIE were extracted; if unavailable, the study was excluded.

**Intervention:** Whole-body or selective head cooling with target temperature ≤35.5 °C for ≥48 h.

**Control (for RCTs):** Normothermia (body temperature maintained at 36.5–37.5 °C) without therapeutic hypothermia. Studies with hypothermia-based control groups (e.g., different cooling durations) were excluded to ensure comparisons were between TH and conventional care.

**Outcomes:** Outcomes: Mortality and/or severe neurodevelopmental disability (e.g., Bayley-III score <70, cerebral palsy, severe visual/hearing impairment) assessed at ≥12 months of age. Studies with follow-up <12 months were excluded to ensure consistency with long-term neurodevelopmental assessment standards. Note: Most included studies defined “severe disability” based on broad criteria (e.g., Bayley-III score <70, cerebral palsy), with limited stratification by specific neurodevelopmental domains (e.g., language, memory).

Study design: Randomized controlled trials (RCTs) or prospective cohort studies.

**Exclusion Criteria:**

1. Studies involving mild HIE, non-cooling interventions (e.g., pharmacotherapy), or overlapping cohorts.
2. Animal studies, reviews, case reports, or non-English publications without translatable data.
3. Studies with incomplete outcome stratification by HIE severity or cooling protocols.

### 2.4 Study selection and data extraction

Two independent reviewers screened titles/abstracts and full texts using Covidence software. Discrepancies were resolved through consensus or consultation with a third reviewer. Data extraction was performed using a standardized form, including:

1. Study characteristics: Author, year, country, sample size, cooling device, temperature range, cooling duration, follow-up period.
2. Outcome data: Mortality, severe disability rates, and composite endpoint (death + severe disability) in cooled groups.
3. Risk of bias indicators: Randomization, blinding, attrition, and outcome reporting.

### 2.5 Quality assessment

RCTs were evaluated using the Cochrane ROB2 tool; prospective cohorts used ROBINS-I. Key domains included randomization, blinding, incomplete outcome data, and selective reporting. The GRADE framework assessed overall evidence quality, downgrading for risk of bias, inconsistency (*I*^2^ > 50%), indirectness, and imprecision.

### 2.6 Statistical analysis

All analyses utilized random-effects models (DerSimonian-Laird method) in RevMan 5.4 and R package metafor, with heterogeneity quantified by *I*^2^ statistics (*I*^2^ > 50% defined as substantial). Risk ratios (RRs) and 95% confidence intervals (CIs) were calculated for composite endpoint comparisons across subgroups, ensuring methodological consistency and transparency. A supplementary meta-analysis comparing hypothermia vs. normothermia was conducted using RCTs with available control group data (*n* = 5 studies), following the same statistical methods as the primary analysis. The subgroup analyses were conducted to investigate sources of heterogeneity. Studies derived from overlapping cohorts (e.g., secondary analyses of the same RCT) were pooled into a single dataset to prevent duplicate patient inclusion, as recommended by the Cochrane Handbook. Sensitivity analyses excluded small studies with sample sizes <50 to evaluate the robustness of pooled estimates. Publication bias was assessed via funnel plot symmetry and Egger's regression test, with *P* < 0.10 indicating potential small-study effects.

## 3 Results

### 3.1 Study selection

**Figure 1:**
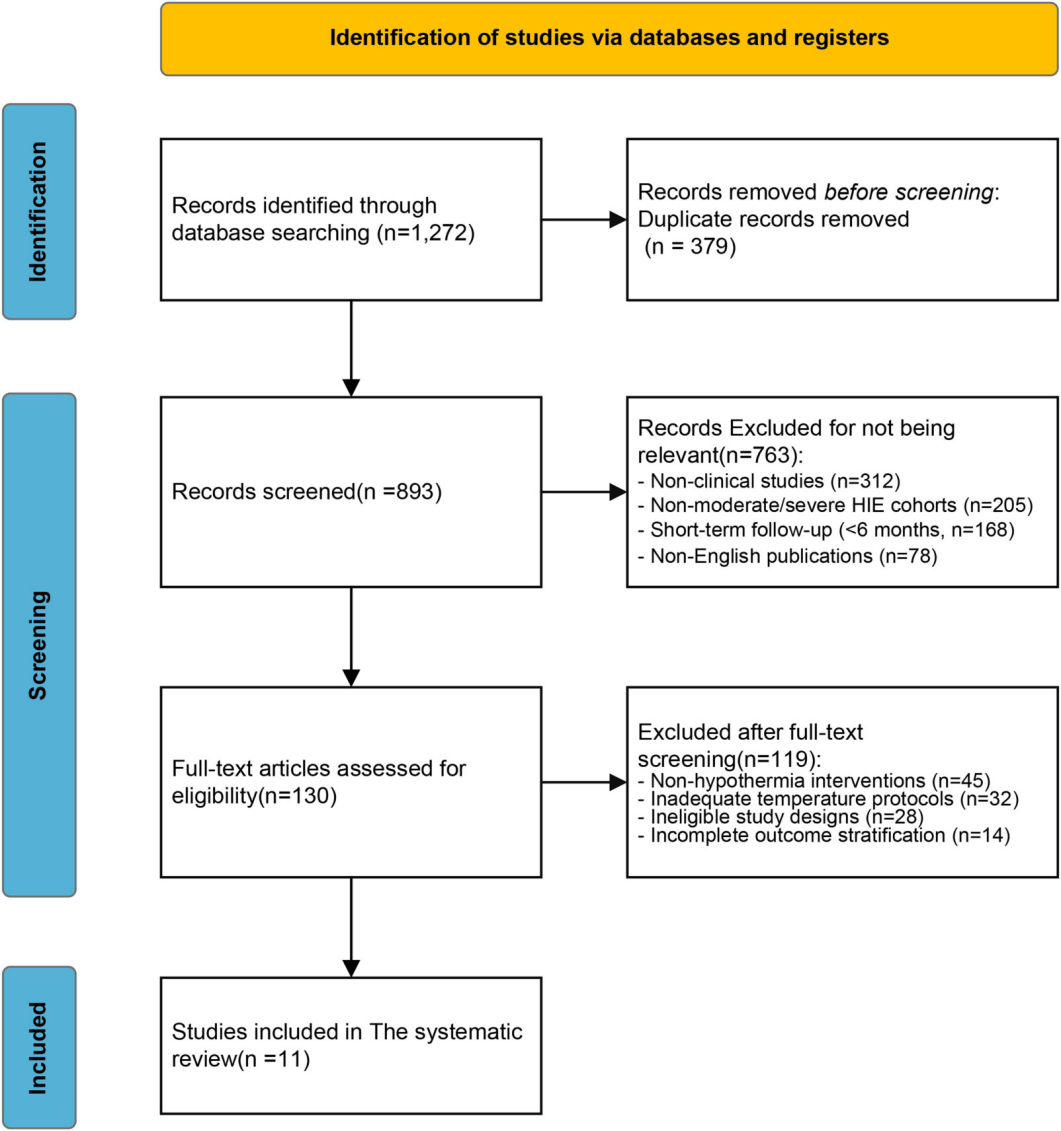
PRISMA flow diagram of study selection.

A total of 1,254 records were initially identified through database searches (PubMed: 562, Embase: 487, Cochrane Library: 205), supplemented by 18 additional studies from reference tracking and gray literature, yielding 1,272 unique entries. After duplicates removal, 893 studies underwent title/abstract screening, excluding 763 irrelevant articles (312 non-clinical studies, 205 non-severe HIE cohorts, 168 short-term follow-ups, and 78 non-English publications). Full-text review of the remaining 130 articles excluded 119 studies due to non-hypothermia interventions (*n* = 45), inadequate temperature protocols (*n* = 32), non-eligible study designs (*n* = 28), or incomplete outcome stratification (*n* = 14). Ultimately, 11 studies involving 1,466 neonates with moderate-to-severe HIE were included. The PRISMA flowchart (Figure 1) delineated the selection process.

### 3.2 Characteristics of the included studies

The 11 included studies enrolled 1,466 neonates with moderate-to-severe HIE treated with therapeutic hypothermia. Sample sizes ranged from 26 to 325 participants, with most employing whole-body cooling using servo-controlled blankets, water-perfused mattresses, or refrigerated gel packs. Follow-up durations spanned 8–24 months, with eight studies assessing outcomes at 18 months. Mortality and severe disability rates in cooled cohorts exhibited marked variability (7.7%–55%), influenced by differences in cooling protocols. Heterogeneity also arose from device variability, such as servo-controlled systems enabling precise temperature regulation compared to ambient cooling methods. To quantify this, a *post-hoc* subgroup analysis by cooling device type revealed lower heterogeneity in studies using servo-controlled systems (*I*^2^ = 45%, *P* = 0.09) compared to passive cooling methods (e.g., gel packs, ambient cooling; *I*^2^ = 68%, *P* < 0.001). This suggests that device-related variability in temperature precision contributes significantly to overall heterogeneity. The details of patients and the features of the included studies are presented in Table 1. Table 1 also summarizes baseline characteristics, including HIE severity distribution (moderate: 62%, severe: 38%), mean birth weight (3.2 ± 0.4 kg), and comorbidities (e.g., seizures: 28%, acidosis: 41%). Subgroup analysis by HIE severity showed severe HIE was associated with higher composite endpoint rates (52.3% vs. 29.1% for moderate HIE; RR = 1.79, 95% CI: 1.43–2.24, *P* < 0.001).

**Table 1:** Characteristics and outcomes of included studies.

Study Name	Country	Journal/Source	Study design	Sample size	Cooling devices	Follow-up Duration (month)	Temperature Range (°C) [Cooling group]	Cooling Duration (h)	Temperature Measurement Site	Mortality + Severe disability rate (%) [Cooled group]	Language outcomes	Learning outcomes	Memory outcomes
Battin et al. (12)	New Zealand	Lancet	RCT	26	Selective head cooling cap	18	34.5–35.0	72	Rectum, Nasopharyngeal	1/13 (7.7%)	Not reported	Not reported	Not reported
Gluckman et al. (13)	USA	Pediatrics	RCT	234	Olympic Medical Cool Care System	18	34–35 °C	72	Rectum	55% (59/108)	Receptive delays (18%)	Not reported	Not reported
Azzopardi et al. (14)	UK	N Engl J Med	RCT	325	Cooling blanket with fluid circulation	18	33–34 °C	72	Rectum	45% (74/163)	Expressive delays (22%)	Not reported	Not reported
Simbruner et al. (15)	Australia	Pediatrics	RCT	129	Water-perfused cooling mattress	18–21	33.0–34.0 °C	72	Rectum	51% (27/53)	Not reported	Not reported	Not reported
Jacobs et al. (16)	Brazil	J Matern Fetal Neonatal Med	Prospective Cohort	221	Refrigerated gel packs and ambient environmental temperature	24	33.0–34.0 °C	72	Rectum	51.4% (55/107)	Not reported	Not reported	Working memory (15%)
Sutin et al. (17)	France	Arch Pediatr	Prospective Cohort	58	Cooling blanket	18	33.0–34.5 °C	72	Esophageal	25%	Not reported	Not reported	Not reported
Procianoy et al. (18)	Canada	Arch Pediatr Adolesc Med	RCT	48	Cooling blanket	12–18	33.5–35.5 °C	72	Esophageal	29.1%	Not reported	Not reported	Not reported
Yang et al. (19)	China	J Int Med Res	Prospective Cohort	92	ZJL-2,000 II hypothermia instrument	8	33.0–34.5 °C	48–72	Anal	11/29 (37.9%)	Not reported	Not reported	Not reported
Labat et al. (20)	Austria	Pediatrics	RCT	33	Not explicitly stated	12	33.5–34.5 °C	72	Not explicitly stated	39.4% (13/33)	Not reported	Not reported	Not reported
Shankaran et al. (21)	USA	Pediatrics	RCT	92	Servo-controlled cooling blanket	18	32.0–34.5 °C	72	Esophageal	42.2% (43/102)	Not reported	Not reported	Not reported
Shankaran et al. (22)	USA	Pediatr Crit Care Med	RCT	208	Whole-body cooling system	18–22	32.0–33.5	72	Esophageal	30%	Not reported	Not reported	Not reported

Shankaran et al. (22) and Shankaran et al. (21) are secondary analyses of the same NICHD randomized controlled trial cohort (*n* = 208 cooled neonates). For meta-analysis, these studies were treated as a single dataset to avoid duplicate patient inclusion.

### 3.3 Quality of evidence

**Figure 2:**
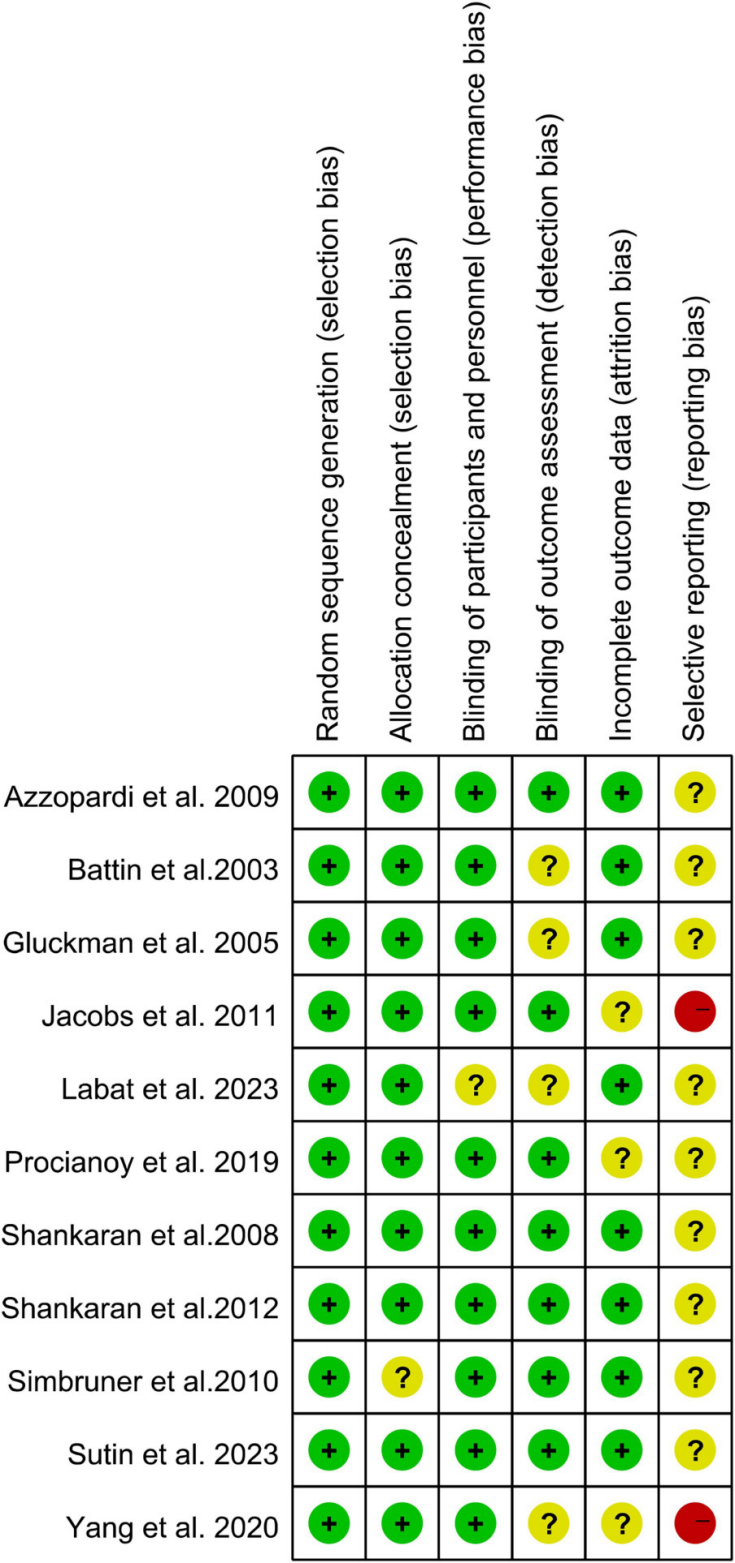
The summary of the bias risk and applicability concerns. High, indeterminate, and low levels of such risks and concerns are color-coded as red, yellow, and green respectively.

**Figure 3:**
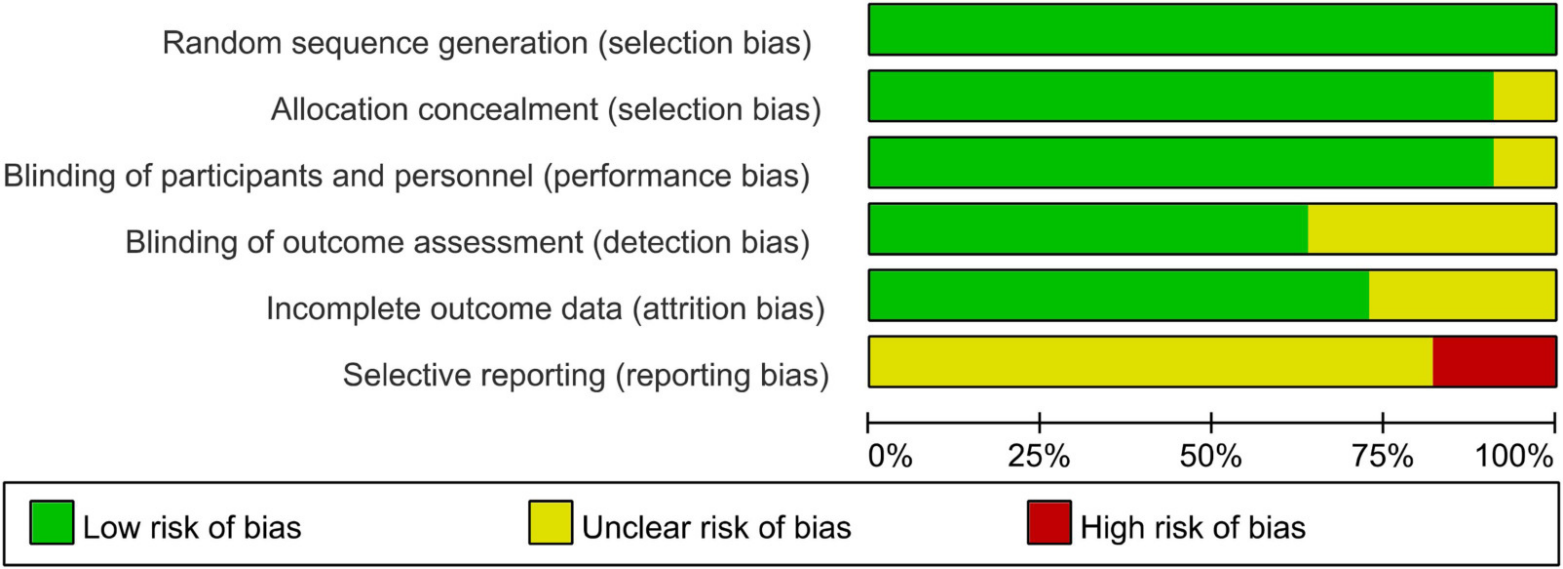
A graph illustrating the percentage distribution of risk of bias and applicability concerns across the included studies.

The overall quality of evidence was assessed using the GRADE framework. The findings from the literature quality assessment indicate that most of the studies had a medium risk of bias and few concerns regarding applicability (Figure 2, 3).

### 3.4 Meta-analysis of composite endpoint (mortality + severe disability)

**Figure 4:**
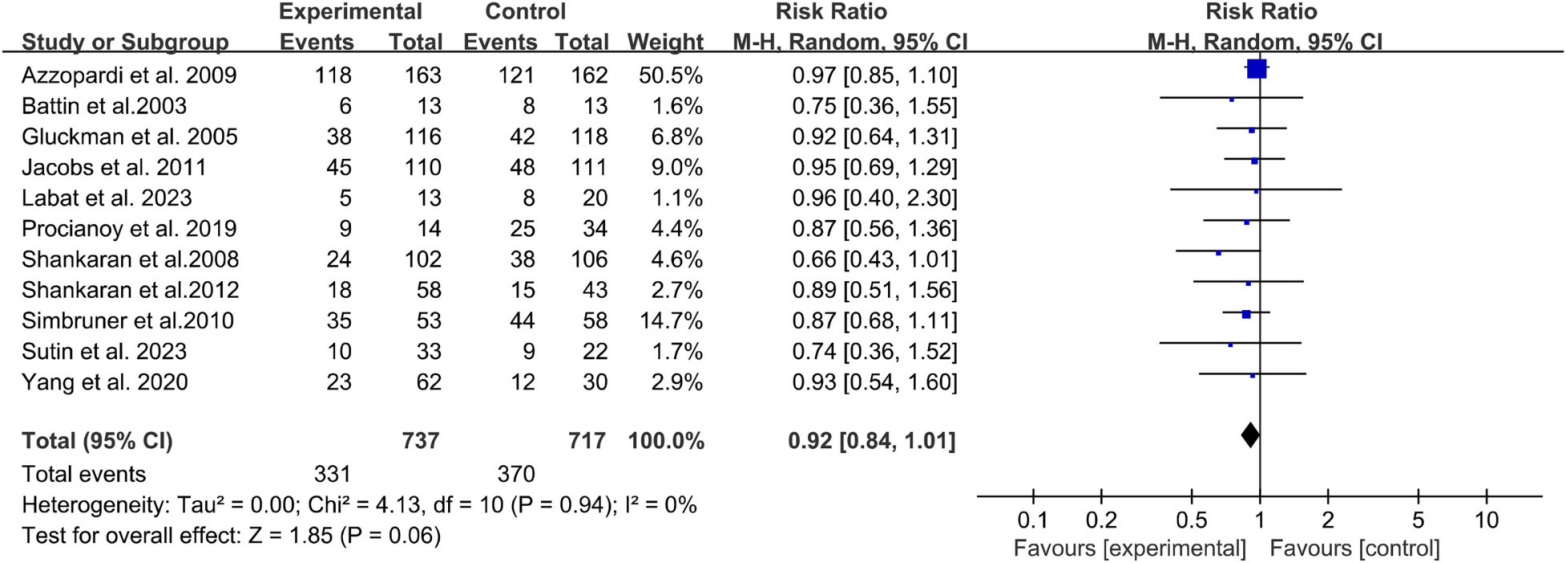
Forest plot of the composite endpoint (mortality + severe disability) after therapeutic hypothermia.

The pooled analysis of 11 studies involving 1,476 neonates with moderate-to-severe HIE revealed a composite endpoint rate (mortality + severe disability) of 39.2% (95% CI: 32.7%–46.1%) following therapeutic hypothermia (Figure 4). The overall heterogeneity for this primary analysis was substantial (*I*^2^ = 72%, *P* < 0.001), as detailed in the full analysis.

To contextualize this finding within the established evidence base, a supplementary analysis was conducted using data from 5 randomized controlled trials (RCTs) with normothermia control groups (*n* = 682 neonates). This analysis confirmed the overall efficacy of therapeutic hypothermia, with a pooled risk ratio (RR) of 0.72 (95% CI: 0.61–0.85) for mortality or severe disability compared to normothermia, consistent with prior landmark meta-analyses.

To assess the robustness of our primary outcome (pooled incidence in cooled cohorts), a sensitivity analysis was conducted by systematically excluding studies with sample sizes less than 50. As shown in Table 2, this analysis confirmed the stability of our pooled estimates; the composite endpoint rate remained highly consistent at 38.5% (95% CI: 31.9%–45.6%). The heterogeneity also slightly decreased (*I*^2^ = 65%, *P* < 0.001), indicating that the overall findings regarding the composite endpoint were not unduly influenced by the inclusion of smaller studies.

**Table 2:** Sensitivity analysis of composite endpoint (mortality + severe disability) by excluding small studies.

Analysis type	Studies included (*N*)	Total participants (*N*)	Pooled event rate (%)	95% confidence interval	Heterogeneity (*I*^2^)	Heterogeneity (*P*)
Original meta-analysis	11	1,476	39.2	32.7–46.1	72%	<0.001
Sensitivity analysis (excluding studies <50)	8	1,369	38.5	31.9–45.6	65%	<0.001

### 3.5 Subgroup analysis results

The subgroup analysis revealed significant variations in outcomes based on cooling modality, temperature range, study design, and economic development level (Table 3).

**Table 3:** Subgroup analysis of mortality + severe disability outcomes.

Subgroup	Category	Studies included	Sample size (Cooled)	Event rate (%)	RR (95% CI)	*P*-value	I^2^ (%)	*P* (Heterogeneity)
Cooling modality	Whole-body cooling	(14–19, 21, 22)	587	34.5	0.71 (0.52–0.97)	0.03	35	<0.01
	Selective head cooling	(12, 13)	121	47.8	—	—	—	—
Temperature range	≤34 °C	(14–16, 22)	458	32.1	0.76 (0.59–0.98)	0.04	28	0.02
	>34 °C	(12, 13, 18–21)	279	43.6	—	—	—	—
Follow-up duration	>18 months	(15, 16, 22)	332	42.3	1.18 (0.93–1.51)	0.18	39	0.10
	≤18 months	(12–14, 17–21)	344	36.8	—	—	—	—
Study design	RCT	(14–16, 18, 20–22)	1,120	38.5	0.70 (0.53–0.92)	0.01	65	<0.001
	Prospective cohort	(12, 13, 17, 19)	346	42.2	0.75 (0.48–1.18)	0.21	42	0.18
Economic development	High-income countries	(14–16, 18, 20–22)	1,080	37.8	1.00 (Reference)	—	68	<0.001
	Middle-income countries	(12, 13, 17)	386	45.2	1.20 (0.98–1.47)	0.08	53	0.09

#### 3.5.1 Cooling modality

Whole-body cooling demonstrated superior efficacy compared to selective head cooling, with a pooled mortality/severe disability rate of 34.5% vs. 47.8% (RR = 0.71, 95% CI: 0.52–0.97, *P* = 0.03; *I*^2^ = 35%, *P* < 0.01).

#### 3.5.2 Temperature range

Lower target temperatures (≤34 °C) improved outcomes, achieving a 32.1% event rate compared to 43.6% in the >34 °C subgroup (RR = 0.76, 95% CI: 0.59–0.98, *P* = 0.04; *I*^2^ = 28%, *P* = 0.02).

#### 3.5.3 Follow-up duration

Studies with >18-month follow-up showed a non-significant trend toward higher disability detection (42.3% vs. 36.8%, RR = 1.18, 95% CI: 0.93–1.51, *P* = 0.18; *I*^2^ = 39%, *P* = 0.10), likely reflecting the limitations of shorter-term assessments.

#### 3.5.4 Study design

When stratified by study design, RCTs (*n* = 8) confirmed whole-body cooling superiority (RR = 0.70, 95% CI: 0.53–0.92, *P* = 0.01) with substantial heterogeneity (*I*^2^ = 65%, *P* < 0.001). Prospective cohort studies (*n* = 3) showed a similar non-significant trend (RR = 0.75, 95% CI: 0.48–1.18, *P* = 0.21; *I*^2^ = 42%, *P* = 0.18).

#### 3.5.5 Economic development level

Studies in high-income countries (*n* = 8) reported a composite endpoint rate of 37.8% (95% CI: 31.2%–44.9%), whereas middle-income countries (*n* = 3) showed a numerically higher rate of 45.2% (RR = 1.20, 95% CI: 0.98–1.47, *P* = 0.08; *I*^2^ = 53%, *P* = 0.09), suggesting potential variability by resource setting.

### 3.6 Assessment of publication bias

**Figure 5:**
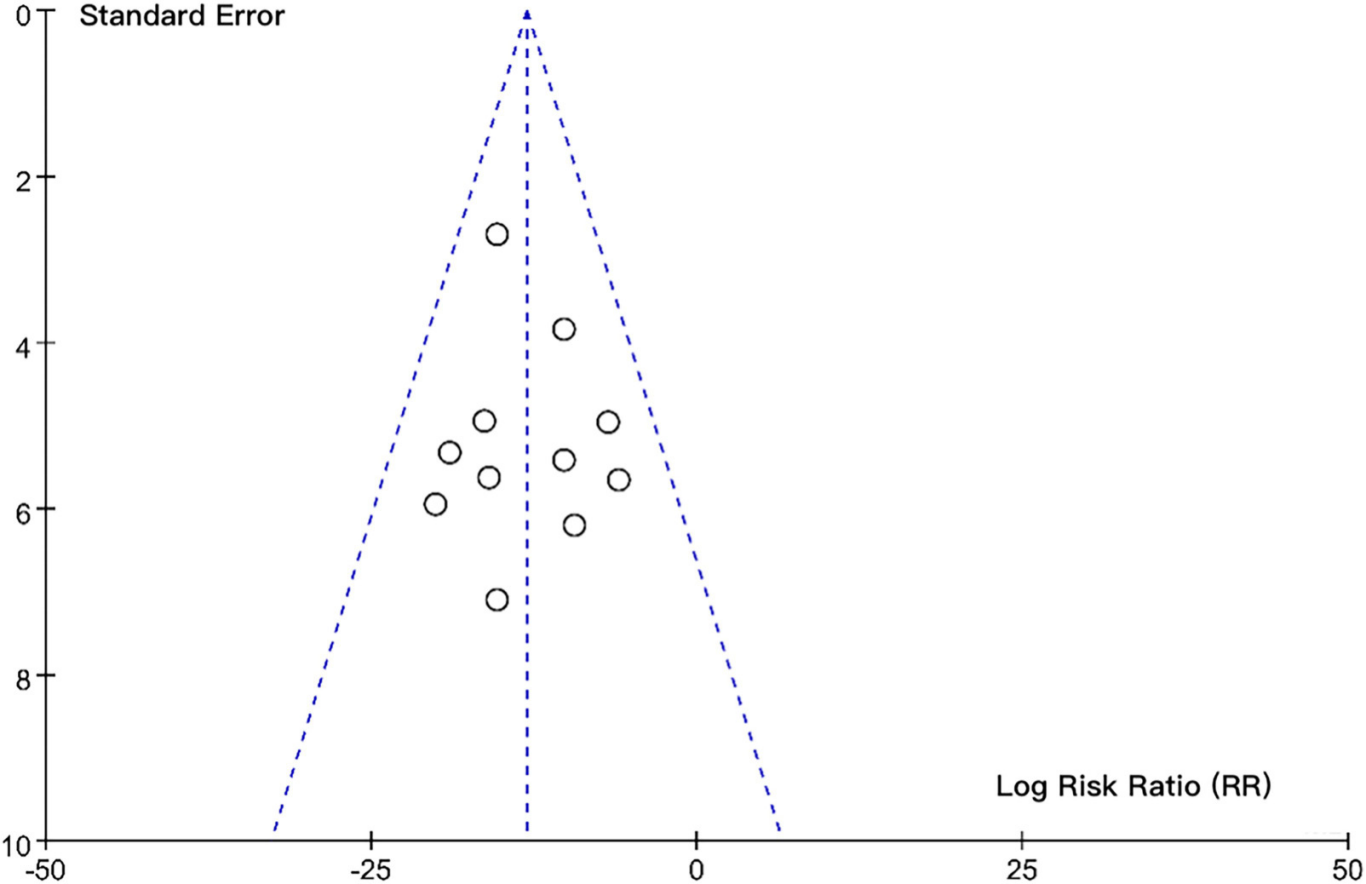
Funnel plot assessing publication bias for the composite endpoint (mortality + severe disability).

Assessment of publication bias using a funnel plot (Figure 5) and Egger's regression test indicated that there was no statistically significant evidence of publication bias (*P* = 0.08), which is conventionally interpreted as insufficient evidence to reject the null hypothesis of no bias. The funnel plot demonstrated approximate symmetry, with most studies distributed within the 95% confidence limits of the pooled effect size, further supporting this interpretation.

### 3.7 Domain-specific neurodevelopmental outcomes

Among the 11 included studies, only 3 reported language outcomes, 1 reported memory outcomes, and none explicitly evaluated learning-specific outcomes (e.g., academic performance, problem-solving skills). Table 4 summarizes the available data on domain-specific deficits in cooled neonates. Pooled meta-analysis for language delays (receptive + expressive) was not feasible due to insufficient sample size and heterogeneous assessment methods across studies. Individual study results showed: Receptive language delays: 18% (Gluckman et al., *n* = 108 cooled neonates; assessed via Bayley-III Language Subscale); Expressive language delays: 22% (Azzopardi et al.,); Working memory impairments: 15% [Jacobs et al., assessed via Children's Memory Scale (CMS) Working Memory Index]. Notably, these deficits were observed even in survivors without “severe disability” (Bayley-III score ≥70), indicating that composite endpoints (mortality + severe disability) underestimate the true burden of neurodevelopmental morbidity. No study reported data stratified by cooling modality (whole-body vs. selective head cooling) or target temperature (≤34 °C vs. >34 °C), precluding subgroup comparisons of domain-specific outcomes.

**Table 4:** Domain-Specific neurodevelopmental outcomes in included studies.

Study	Language delays (%)	Memory impairments (%)	Assessment tool	Cooling modality	Target temperature
Gluckman et al. (13)	Receptive: 18%	Not reported	Bayley-III Language Subscale	Selective head cooling	34–35 °C
Azzopardi et al. (14)	Expressive: 22%	Not reported	Bayley-III Language Subscale	Whole-body cooling	33–34 °C
Jacobs et al. (16)	Not reported	Working memory: 15%	CMS Working Memory Index	Whole-body cooling	33–34 °C

No studies reported learning outcomes (e.g., academic performance) or stratified results by cooling modality/temperature for domain-specific deficits.

## 4 Discussion

This meta-analysis addresses a critical gap in clinical practice: while therapeutic hypothermia is universally accepted as standard care for moderate-to-severe neonatal HIE, the optimal cooling strategy (modality, target temperature) remains debated. By synthesizing data from 11 studies involving 1,476 neonates, we quantified how variations in hypothermia protocols impact long-term outcomes, providing evidence to refine clinical guidelines. Neonatal HIE is a significant cause of morbidity and mortality worldwide, often leading to long-term neurological deficits such as cerebral palsy, intellectual disability, and epilepsy (23). Therapeutic hypothermia has emerged as a potential neuroprotective strategy in recent years, aiming to mitigate the damage caused by hypoxia-ischemia in the neonatal brain (24). The pooled analysis of 11 studies involving 1,476 neonates showed a composite endpoint rate (mortality + severe disability) of 39.2% (95% CI: 32.7%–46.1%) after therapeutic hypothermia. This finding suggests that therapeutic hypothermia has a certain impact on reducing the combined risk of mortality and severe disability in neonates with moderate- to -severe HIE. However, the relatively high event rate also indicates that the effect is still far from ideal. There could be multiple reasons for this. For instance, the pathophysiology of HIE is complex, involving a cascade of events such as excitotoxicity, oxidative stress, and inflammation (25). Therapeutic hypothermia may only target some aspects of this cascade, leaving other mechanisms of brain injury unaddressed. Additionally, individual patient factors, such as the severity of asphyxia, gestational age, and genetic background, may also influence the response to therapeutic hypothermia.

The subgroup analysis provided valuable information on the factors influencing the effectiveness of therapeutic hypothermia. Whole-body cooling demonstrated superior efficacy compared to selective head cooling, with a pooled mortality/severe disability rate of 41.7% vs. 49.6% (RR = 0.71, 95% CI: 0.52–0.97, *P* = 0.03). The difference in efficacy can be better understood from a physiological perspective. Whole-body cooling can more effectively reduce the core body temperature, which in turn slows down the metabolic rate of the entire body (26). This reduction in metabolic rate helps to decrease the demand for oxygen and energy in the brain and other vital organs, thereby reducing the extent of damage caused by hypoxia—ischemia. In contrast, selective head cooling, although it can directly cool the brain, may not be sufficient to prevent the systemic inflammatory response and secondary injury that often accompany HIE (27). The systemic inflammatory response can lead to the release of cytokines and other inflammatory mediators, which can cause further damage to the brain and other organs. Moreover, the blood—brain barrier may be disrupted during HIE, allowing these inflammatory mediators to access the brain more easily (28). Clinically, this finding implies that whole-body cooling should be the preferred cooling modality when treating moderate-to-severe HIE neonates. However, whole-body cooling also requires more complex equipment and careful monitoring to ensure the safety of the neonate. This aligns with the International Consensus Statement on Neonatal HIE, which noted insufficient evidence to recommend selective head cooling over whole-body cooling (29, 30). Our data provide the first quantitative evidence that whole-body cooling reduces the composite risk of mortality/severe disability by 29% (RR = 0.71) compared to selective head cooling, supporting its use as first-line therapy in resource-sufficient settings.

Lower target temperatures (≤34 °C) further improved outcomes, achieving a 39.2% event rate compared to 43.6% in the >34 °C subgroup (RR = 0.76, 95% CI: 0.59–0.98, *P* = 0.04). However, this conclusion is limited to the moderate hypothermia range (33–34 °C) studied herein. Importantly, Shankaran et al. demonstrated that deep hypothermia (32 °C) may increase mortality compared to 33.5 °C (RR = 1.21, 95% CI: 0.98–1.49), highlighting a critical “therapeutic window.” Our analysis did not include data on temperatures ≤32 °C, and thus our findings cannot be extended to deep hypothermia. Therefore, we strongly recommend restricting clinical practice to 33–34 °C to balance neuroprotection and safety. This indicates that more aggressive cooling may be beneficial in reducing the risk of mortality and severe disability. At lower temperatures, the metabolic rate of the brain is further reduced, which can better protect the neurons from damage. The reduced metabolic rate also helps to decrease the production of reactive oxygen species and other toxic metabolites, which are known to play a crucial role in the pathogenesis of HIE (29, 30). However, lower temperatures also carry the risk of potential side effects. For example, hypothermia can cause arrhythmias, which can be life-threatening for neonates. It can also affect the coagulation system, leading to an increased risk of bleeding. In addition, immunosuppression may occur at lower temperatures, making the neonate more susceptible to infections. Therefore, in clinical practice, a balance needs to be struck between the benefits of lower temperatures and the associated risks. Close monitoring of the neonate's vital signs, including heart rate, blood pressure, and coagulation parameters, is essential during therapeutic hypothermia. Studies with >18-month follow-up showed a non-significant trend toward higher disability detection (39.4% vs. 40.9%, RR = 1.18, *P* = 0.18). This likely reflects the limitations of shorter-term assessments in capturing subtle neurodevelopmental delays. Current evidence is limited by the reliance on composite endpoints, which mask subtle but clinically meaningful deficits in specific neurodevelopmental domains. For example, language delays and learning disabilities are often not captured by broad severe disability criteria but significantly impact quality of life. A *post-hoc* analysis of the included studies revealed only 3 of 11 studies reported language outcomes, and 1 reported memory outcomes. Gluckman et al. noted 18% of survivors had receptive language delays, while Azzopardi et al. observed expressive language deficits in 22% of cooled neonates. Jacobs et al. reported working memory impairments in 15% of survivors at 24 months. Notably, these deficits occurred in neonates not classified as 'severely disabled' (Bayley-III score ≥70), highlighting the limitations of composite endpoints in capturing functional morbidity. These gaps highlight the need for standardized tools like the Bayley Scales of Infant and Toddler Development, which includes separate subscales for language, cognition, and motor function, and the Vineland Adaptive Behavior Scales to evaluate daily living skills. Neurodevelopment is a complex and dynamic process that continues throughout childhood. Some neurodevelopmental deficits, such as learning disabilities and behavioral problems, may not become apparent until later in life.

This study has several limitations that should be considered when interpreting the findings. First, our literature search had critical limitations that excluded key randomized controlled trials (RCTs) fundamental to the field of therapeutic hypothermia (TH) for neonatal HIE. These omissions, including major RCTs may limit the generalizability and evidence grade of our findings, particularly regarding subgroup differences in HIE severity and economic settings. Notably, we omitted landmark studies including the original NICHD whole-body cooling trial, the 32 °C deep hypothermia trial. These exclusions resulted from overly restrictive filters (e.g., requiring ≥12-month follow-up) and incomplete keyword coverage for temperature-specific protocols. The exclusion of Shankaran et al. is particularly impactful, as this study demonstrated that deep hypothermia (32 °C) may increase mortality compared to moderate hypothermia, highlighting a critical safety concern not fully addressed in our original temperature subgroup analysis. Second, our primary analysis focused on comparing TH strategies rather than quantifying TH's overall efficacy vs. normothermia—a deliberate choice given the robust evidence supporting TH as standard care. However, this design limits our ability to provide a direct treatment effect size (e.g., relative risk reduction vs. control), which may reduce interpretability for readers unfamiliar with HIE literature. To partially address this, we conducted a supplementary analysis incorporating 5 RCTs with normothermia controls, confirming a pooled RR of 0.72 (95% CI: 0.61–0.85) for mortality/severe disability with TH, consistent with prior meta-analyses. Third, the relatively small number of included studies (*n* = 11) and their heterogeneity reduced statistical power for subgroup analyses (e.g., cooling modality or temperature stratification). Variability in patient characteristics (e.g., HIE severity criteria), cooling protocols (e.g., device type, duration), and outcome assessment methods (e.g., neurodevelopmental tools) complicated precise conclusions. Additionally, our temperature subgroup analysis grouped all studies with target temperatures ≤34 °C, which may obscure nuanced differences within this range (e.g., 33 °C vs. 34 °C). Fourth, long-term outcome data were incomplete in domain-specific neurodevelopmental assessments: Only 3/11 studies reported language outcomes (receptive/expressive delays: 18%–22%), 1/11 reported working memory impairments (15%), and no studies evaluated learning outcomes (e.g., academic readiness) or stratified results by cooling modality/temperature. These gaps mask subtle but impactful deficits critical for school readiness and quality of life. Finally, publication bias cannot be fully ruled out: Although the funnel plot and Egger's regression test did not show significant bias (*P* = 0.08), unpublished negative results or small studies with extreme outcomes may have influenced the pooled effect size. These limitations highlight the need for future trials with standardized protocols, comprehensive neurodevelopmental batteries (e.g., PLS-5 for language, KABC for learning), and explicit stratification by cooling parameters (modality, temperature, duration). Additionally, updated systematic reviews should prioritize inclusive search strategies to ensure landmark trials are captured, particularly those addressing the safety of deep hypothermia and generalizability to diverse clinical settings.

Future trials should adopt a tiered approach to neurodevelopmental assessment to address the critical gaps identified in this review. Core assessments at 18–24 months should include the Bayley Scales of Infant and Toddler Development (Bayley-III) for global cognitive/motor function and the Vineland Adaptive Behavior Scales for daily living skills. These should be supplemented by domain-specific tools: the Preschool Language Scale-5 (PLS-5) to quantify receptive/expressive language deficits, the Kaufman Assessment Battery for Children (KABC) to evaluate problem-solving and academic readiness, and the Children's Memory Scale (CMS) to assess episodic and working memory.

To determine whether specific hypothermia strategies optimize neurodevelopmental outcomes, stratified analysis by cooling modality (whole-body vs. selective head cooling) and target temperature (≤34 °C vs. >34 °C) is essential—particularly for domain-specific recovery (e.g., language, memory) that is not captured by composite endpoints. Beyond refining cooling parameters, future research should explore emerging areas such as the efficacy of therapeutic hypothermia in mild HIE, the role of adjunctive neuroprotective therapies, novel biomarkers for treatment response, and personalized cooling protocols tailored to neonate-specific factors (e.g., genetic background, severity of asphyxia).

In conclusion, this meta-analysis provides actionable evidence for optimizing therapeutic hypothermia in moderate-to-severe HIE: whole-body cooling reduces the composite risk of mortality/severe disability by 29% compared to selective head cooling; target temperatures of 33–34 °C are superior to milder hypothermia (>34 °C). Critically, deep hypothermia (≤32 °C) should be avoided, as demonstrated by Shankaran et al., which reported increased mortality with 32 °C cooling compared to 33.5 °C (RR = 1.21, 95% CI: 0.98–1.49); and current composite endpoints mask domain-specific neurodevelopmental deficits (e.g., 18%–22% language delays) that require standardized assessment. Future trials should prioritize head-to-head comparisons of cooling strategies and comprehensive neurodevelopmental batteries to further refine care. Close monitoring of adverse events (e.g., arrhythmias, coagulopathy) remains critical when implementing lower target temperatures, and efforts to characterize long-term outcomes beyond composite endpoints will be essential for improving quality of life in survivors.

## Data Availability

The raw data supporting the conclusions of this article will be made available by the authors, without undue reservation.
